# Exploring barriers and solutions in sexual health services: a qualitative study from Iran

**DOI:** 10.1186/s12978-025-02165-0

**Published:** 2025-10-15

**Authors:** Zeinab Hamzehgardeshi, Marzieh Azizi, Jila Ganji, Maryam Farmahini Farahani, Keshvar Samadaee Gelehkolaee

**Affiliations:** 1 Department of Reproductive Health and Midwifery, Sexual and Reproductive Health Research Center, Mazandaran University of Medical Sciences, Sari, Iran; 2 Department of Midwifery, TeMS.C., Islamic Azad University, Tehran, Iran

**Keywords:** Sexual health, Stakeholder, Barriers, Solutions, Healthcare services, Qualitative research

## Abstract

**Background:**

Sexual health and access barriers have become increasingly important, especially given the rising divorce rates and marital dissatisfaction linked to sexual health literacy. This qualitative study aims to explore the barriers and solutions affecting service provision in sexual health clinics (SHCs) in Sari, Iran, from the perspectives of key stakeholders.

**Methods:**

This qualitative study was conducted from July to October 2024 using a conventional content analysis approach. It involved in-depth, semi-structured interviews, and Focus Group Discussions (FGDs) with a diverse group of participants. Data saturation was achieved after engaging with four policymakers, four specialists, 26 clients, and 120 health providers.

**Results:**

The findings reveal three main categories: Challenges, Opportunities, and Solutions. Under Challenges, structural, socio-cultural, professional and organizational, and service-related challenges were identified. Opportunities highlighted university capacities, alignment with national policies, autonomy, and effective leadership strategies. Proposed solutions included resource development, the implementation of evidence-based practices, and de-labeling efforts to reduce the stigma surrounding sexual health.

**Conclusion:**

The study emphasizes the need to enhance sexual health literacy and improve access to sexual health centers in Iran through infrastructure development, educational initiatives, and open dialogue to reduce stigma. Addressing these challenges can better meet the population’s sexual health needs and improve overall health.

## Plain language summary

Sexual health clinics (SHCs) are important because they offer services such as testing and treating sexually transmitted infections (STIs), providing preventive care, and offering support in a welcoming, stigma-free environment. Research shows that sexual health affects many areas of life, including physical health, mental health, relationships, job stability, and lifestyle choices. Because sexual health issues are often stigmatized, it is crucial to address these concerns to improve overall well-being. Understanding and supporting individuals experiencing sexual health problems is important. By examining the challenges and solutions within sexual health services, and by considering cultural expectations, we can develop better strategies and tools to improve quality of life for everyone.

## Background

In recent years, the implementation of population policies aimed at influencing fertility has become a focal point in many societies [1, 2]. Marital dissatisfaction remains a key contributor to divorce, despite efforts to strengthen relationships. Frequently cited reasons include sexual dissatisfaction and the inability of couples to express love and excitement [3, 4]. This trend raises critical concerns about the overall health and quality of sexual relationships within couples, as these can be adversely affected by a lack of sexual health literacy [5, 6]. Globally, the implications of insufficient sexual health education are clear. Between 2015 and 19, there were approximately 121 million unintended pregnancies annually, resulting in a rate of 64 unintended pregnancies per 1,000 women aged 15–49 years, with 61% of these ending in abortion [7]. The global burden of STIs is largely due to longstanding inequalities in resource distribution, particularly in lower- and middle-income countries, which bear the greatest impact. In contrast, higher-income regions have seen significant improvements in HIV (Human Immunodeficiency Virus) and HPV (Human Papillomavirus) prevention due to better access to interventions [8]. Sexual rights violations, lack of access to evidence-based data, and poor implementation of programs further complicate the situation [9]. Recognizing these complexities, the World Association for Sexual Health promotes an interdisciplinary and multisectoral approach to research, program design, and service delivery that fully integrates the connections between sexual health, rights, and pleasure [10]. Studies indicate that enhancing sexual health literacy is an effective strategy for improving overall sexual health [6, 11].

Sexual health clinics (SHCs) play a vital role in addressing sexual and reproductive health challenges by providing essential services such as diagnosing and treating STIs, offering preventive care, and delivering complex clinical support in a stigma-free environment [12]. In recent years, awareness of the services provided by SHCs in Iran has remained limited. Despite substantial research highlighting the sexual and reproductive health needs and challenges, a significant gap remains in understanding the barriers to service provision and potential solutions in these clinics [13, 14]. This study aims to explore barriers and facilitators from the perspectives of key stakeholders, thereby contributing to improving service delivery and accessibility in SHCs. Identifying and addressing these critical factors will better inform strategies to promote sexual health literacy and improve sexual health outcomes in the population.

## Methods

### Study design

The present qualitative study, conducted in Sari (in northern Iran) from July to October 2024, used conventional content analysis to explore the barriers and facilitators of providing services in SHCs from the perspective of stakeholders.

### Study population: characteristics and selection of participants

The target group of this study was policymakers, professionals, and individuals visiting SHCs. Interviews were conducted with four policymakers, three specialists, 26 clients, and 120 healthcare providers, who were selected through purposeful sampling methods to ensure maximum diversity in age, socio-economic level, expertise, and workplace (public or private centers). The inclusion criteria were: Iranian nationality; written informed consent to participate in the research; mental stability during the interview; specialists currently providing services in the SHCs; and policymakers involved in designing and launching the university’s SHCs. Participants who chose not to continue the interview were excluded from the study.

### Data collection

In-depth, semi-structured, face-to-face interviews were carried out to collect data. According to the University of Medical Sciences executive protocol, two approaches are currently being implemented. The first is a team approach led by specialists, including a gynecologist with a pelvic floor fellowship. This team, after the initial interview and screening, refers clients to a psychiatrist, urologist, or sexual and reproductive health specialist as needed. In the second approach, a sexual and reproductive health specialist visits the clients and, after the initial interview and screening, refers them to higher levels of care if necessary. In addition to individual interviews, several focused group discussion (FGD) sessions were conducted with health providers involved in health service packages. An effort was made to invite all health care providers offering sexual and reproductive health services in the county to multiple sessions.

Arrangements for the individual interviews were made in advance through a phone call. An invitation was sent to health providers located in health centers by the university’s Health Vice-Ministers to participate in a training and collaboration workshop, mentioning the title of the research project. A total of 10 FGD sessions were held, each attended by 12 people.

In the beginning, the project was introduced and participants were invited to share their opinions. Their thoughts were gathered regarding the extent and manner of their familiarity with the university’s sexual health clinic, the referral process, and their views about the clinic and its services (during the first hour). Subsequently, a three-hour training session was held on how to refer clients, the types of specialties available at the clinic, and effective communication with clients and their referrals. Meetings for policymakers were primarily conducted at the university. The interviews were carried out by a specialist in sexual and reproductive health, who was also an expert in qualitative research methods, using a semi-structured questionnaire based on the interview guide (see Table 1).

**Table 1:** Interview guide questions for stakeholders (references, experts, policymakers)

Open questions	Probing questions
Please share your experiences about attending the sexual health clinic.	Positive and negative experiences
How did you evaluate the services received in the clinic?	Compared to before starting the clinic, do you have more differences in how you feel or look at sexual health? Please explain
What were your expectations about receiving clinic services?	
How did you get to know the clinic’s location and services?	
How do we inform people better about clinic services?	Please explain more about the other possible methods apart from how you met.
What obstacles did you identify with attending and receiving services from the clinic?	How did you perceive them as obstacles?
What obstacles and facilitators did you face in setting up the clinic?	Please explain more about the reasons for the obstacles.

Probing questions such as “What do you mean?“, “How?“, and “Could you explain more, if possible?” were asked to extract more information from participants and to understand their opinions. Each interview lasted between 40 and 60 min. At the end of the interviews, participants were informed about the possible need for further interviews. Interviews continued until data saturation was reached.

### Data analysis

Conventional content analysis, following the Granheim and Lundman approach, was employed [15]. The recordings were transcribed, and the text was read several times until a general understanding of the interview content was achieved. This process involved recounting direct quotes from participants, labeling the text, and categorizing it, which led to the extraction of new categories. The entire text was considered the unit of analysis, while shorter parts-such as phrases, sentences, or paragraphs-that had meaning related to the research question were regarded as meaning units. Each meaning unit was condensed into denser meaning units while preserving its original meaning, and then these units were coded. The codes were classified into categories and subcategories based on their similarities and differences. Finally, according to the underlying meanings of the statements, main categories were extracted. MAXQDA 10 software was used for data analysis.

### Trustworthiness

Lincoln and Guba’s criteria, which include credibility, dependability, transferability, and confirmability, were used to determine the rigor of the qualitative results [16]. In addition, prolonged interaction with the data, member checking, and maximum diversity in selecting participants contributed to the data’s credibility. More than one research team was involved in the data analysis procedure to enhance the dependability of the results. For this reason, the first author collected and analyzed the data. A research expert team of two experts familiar with qualitative research conducted an external review to ensure an external check. All study steps were recorded and reported in detail for future research, thus ensuring compliance. Finally, transferability was enhanced through thick description.

### Ethical considerations

The present study was conducted in accordance with the Declaration of Helsinki and is derived from a research project approved by the Ethics Committee of Mazandaran University of Medical Sciences (ethics code: IR.MAZUMS.REC.1403.18853). After explaining the study’s objectives and ensuring the confidentiality of participants’ information, both verbal and written informed consent were obtained for recording the interviews. The participants were assured that the interviews would be deleted after data extraction. Interviews were conducted with clients in a special room in the clinic, maintaining privacy and confidentiality. In addition, participants’ right to withdraw from the study without consequences was considered and explained.

## Results

Data saturation was achieved after conducting 10 focus group discussions (FGD) involving 120 health providers and individual interviews with four policymakers, four specialists, and 26 individuals. The age of the participants ranged from 17 to 64 years (Table 2). The results are presented within three main categories (challenges, opportunities, solutions), 11 subcategories, and 25 sub-subcategories (Table 3).

**Table 2:** Demographic characteristics of the participants

Type of activity	Expertise	Age (years)
Specialist	Sexual and reproductive health, urologist, psychiatrist	40–50
Policymaker	Vice President of Education, Director of the Clinic, Chairman of the Executive Board, Vice President of Treatment	45–60
People	Student, employee, freelancer, housewife	17–64
Health Provider	Midwife, family health, psychologist, nurse, counselor	27–54

**Table 3:** Categories and subcategories extracted from the interview

Categories	subcategories	Sub-subcategories	Codes
Challenges	Structural challenges	Access to services	- Difficult access to specialists - Referral system flaw - Clinic days do not match the population
		Privacy and confidentiality	- Inappropriate place for sexual counselin - Failure to meet the standards set for youth-friendly centers - Concerns about data security
		Structural space	- Unpleasant appearance of the room and waiting room
	Socio-cultural challenges	Awareness and education	- Lack of awareness at the micro and macro levels - Lack of proper training Lack of awareness of other specialists about the clinic’s services
		Communication skills	- Weakness in experts’ skills in communicating with clients - Couples’ lack of skills in identifying and expressing sexual problems
		Discrimination	- providing services to gender minorities
		Stigma	- Worry about being seen - Concerns about being included in the case file - A special look at sexual minorities
	Professional and organizational challenges	Specialist	- Lack of adequate support from reproductive health professionals - Conflict of interest between specialists - Lack of interdisciplinary coordination and collaboration - Lack of trained specialists
		Workload	- High workload for healthcare workers
	Service challenges	Defects in service packages	- Defects in existing service packages - Failure to comply with service levels
		Culture-based national protocol	- Lack of national consultation protocols - Designing cultural and evidence-based protocols
		Insurance problems	- High cost of treatment in private centers - These services are not defined in the book Relative Values ​​for Reproductive Health Professionals
Opportunities	University capacities	Clinic location	- Geographical location - Access to academic resources
		Specialties	- Specializations available at the university - Integrated services - Professional competency audit
	Alignment with the country’s macro policies	Educational, research, and cultural policies	- Alignment with academic goals - Coordination of the vice- Ministers - Empowering professors
		Health and medical policies	- Compliance with national programs - Supporting the implementation of projects
	Autonomy	Diverse approaches	- Therapeutic approach - Preventive approach with counseling and screening services
		Innovation in services	- New services - Providing integrated services
		Attention to the needs of the community	- Identifying indigenous needs - Responding to social challenge - Reasonable cost
	Leadership strategies	Management support	- Resource allocation - Suitable setting
		Innovation support	- Support for new ideas - Solidarity of the Vice-Ministers
Solutions	Resource development	infrastructure	- Developing specialized sexual counseling centers - Referral system based on service levels - Designing a suitable computer system to maintain data confidentiality
		Human	- Promoting sexual health literacy - Training of specialists - Returning the health care worker to the midwife (optimal use of expertise) - Empowering and improving communication skills - Collaboration between specialists - Advertising through national media and social networks
	Implementing best evidence	Research activities	- Design, implementation and evaluation of evidence-based national protocols
	De-labeling and de-stigmatization	Educational and cultural activities	- Culture-based content production - Integration into the basic service package - Introducing positive experiences of people in the media - Evaluation, monitoring and publication of results in the media - Promoting dialogue and knowledge translation - Launching HIV test campaigns - Application design

### Challenges

#### Structural challenges

The results show several challenges that impede access to SHCs for the general population, including youth. Difficult access to specialists, a flawed referral system, Physical space, and a mismatch between clinic days and the population served highlighted the challenge of accessing services. As the following statement by a specialist illustrates:

*Sometimes*,* when I am talking to clients*,* the secretary knocks on the door and comes in because they do not know that this room is different from the other rooms in the clinic. They should have been trained beforehand. (Specialist14).*

*The lack of physical facilities is one of the obstacles we face in our work. We need to eliminate clutter*,* unpleasant colors*,* and any feelings of discomfort from the environment. The design of the signboard should not be overly elaborate*,* but it should be inviting enough to make patients feel comfortable. Additionally*,* there is currently no effective computer system. (Specialist10)*.

#### Socio-cultural challenges

Lack of awareness and education at various levels, ineffective communication skills, discrimination, and stigma were prominent challenges in this category. The following discussion will further clarify this topic:

*Cultural taboos*,* both at the family and management levels*,* have made it difficult for us therapists to work. Some socio-cultural regulations prevent us from achieving the best therapeutic results and reduce our motivation. (Specialist10)*.

A different specialist continues by stating: *It appears that after two years*,* other specialists are still unaware of the existence of a sexual clinic in this complex. Additionally*,* the community lacks sufficient information about it. (Specialist 14)*

*I went to another center at the university*,* but they told me that my wife had to come along too*,* which made me leave right away. I don’t want her to know anything about this*,* and I’ve been seeking treatment for a long time. If she finds out*,* I’ll be in a terrible situation. (client 7).*

Another client from a sexual minority expressed, *“Social stigma and moral judgments prevent many individuals*,* particularly sexual minorities*,* from seeking professional help”. Many others are like us*,* and few have families that support them.” (Client 9)*.

#### Professional and organizational challenges

This subcategory highlights factors related to specialists, including financial interests, conflicts of interest, lack of collaboration, and high workloads.

One specialist noted, “*The rights and salaries of specialists should be considered based on their specialization*,* educational level*,* and time commitment to ensure they are motivated to attend consistently and punctually.” (Specialist 11)*.

Midwives mentioned in the FGD: *“Since we have transitioned away from our roles as midwives and are now working as healthcare providers*,* we have not had the opportunity to screen for and refer patients with sexual health issues.”*

#### Service challenges

Along with insurance-related issues, the lack of a culture-based service package was evident in this subcategory.

*The benefits of specialists are not guaranteed*,* as the referral system and insurance companies are still unfamiliar with these services (Specialist 13).*

### Opportunities

The opinions of certain clients and the majority of policymakers in this area are categorized into four subcategories: university capacities, alignment with the country’s macro policies, autonomy, and leadership strategies.

#### University capacities

All subcategories were reflected in this expert’s statements. *While there were some minimum requirements*,* the collaboration among colleagues was robust*,* and the university officials approached the situation with seriousness. Establishing the clinic in two different locations is a significant positive aspect. Everyone’s attitude was optimistic*,* and there was much less resistance than we had anticipated. Often*,* the limitations we encounter are merely in our minds. (Specialist 10)*

#### Alignment with the country’s macro policies

Most managers emphasized the statement, “*It aligns with the population policies.” They noted that when it is included in the country’s macro policies*,* it receives full support. Additionally*,* the university president expressed interest in this matter. (specialist 11*,* 12)*

#### Autonomy

Specialists and policymakers noted that establishing two clinics, each employing different approaches, yields a wide range of benefits. *The integration of services*,* facilitated by having relevant specialists present on the same day at one clinic*,* represents a novel approach. The two clinics were designed with distinct management strategies to evaluate success and satisfaction. (Policymaker 13)*

#### Leadership strategies

One opportunity was to implement effective and timely leadership strategies. *Good resources were allocated from headquarters*,* and colleagues with the required expertise were called upon*. *(Policymaker13)*

*A platform for interdisciplinary collaboration was established and quickly gained momentum as an innovative solution addressing a fundamental societal need. (Policymaker 14)*

### Solutions

Solutions emerged from the discussions as participants enthusiastically shared suggestions based on their experiences. The recommendations include resource development, implementation of best practices, and the promotion of de-labeling and de-stigmatization.

#### Resource development

Providing resources is one of the most important issues to address in advancing the goals of an innovative project. *Specialized centers must be developed*,* along with information technology systems to ensure confidentiality and an effective referral system. (specialist11*,* FGD)*

*Whenever I asked about a fee*,* I was informed that it was a new field and that it wasn’t defined in the book on relative service values. As a result*,* insurance does not provide coverage for reproductive health specialists. (specialist11*,* 15)*

The midwives in the group discussion shouted in unison. *They noted that the service environment in the health centers is insufficient and that clients’ privacy is not respected. Additionally*,* they noted that they do not have the opportunity to focus on their primary role as midwives. Instead*,* they are categorized as healthcare providers serving all age groups*,* which diverts their attention away from women’s health.(FGD)*.

#### Implementing best evidence

Sexual health clinic services are among the most specialized in healthcare provision, both because of their specific focus and the taboo nature surrounding them. *It is essential to incorporate cultural adaptation throughout the entire process*,* from design to evaluation. This involves implementing a national protocol informed by cultural considerations. (specialist13*,* FGD).*

*It should be extracted by researching the clients’ problems. Long before the clinic was launched*,* there were strict rules regarding its title; however*,* in practice*,* the clinic’s name did not attract any objections from the community. (specialist13)*

#### De-labeling and de-stigmatization

The words “taboo,” “stigma,” “label,” and “cultural adaptation” were frequently mentioned at both the beginning and the end of each interview. *It would have been better if we had created content simultaneously with the launch of the clinic and made it accessible to the community through local and social media. This approach would have facilitated a space for dialogue. (Specialist 12)*

*“I believe that sharing stories of individuals who have had positive experiences at the clinic in the media can help break down taboos*,*” said the client.*

Overall, experiences show that if fully calculated and evidence-based, any new step toward culture-building will have beneficial consequences, even if initially difficult.

## Discution

In this qualitative study, we aimed to understand the experiences of stakeholders involved in establishing a sexual health clinic as a new initiative for maintaining and promoting sexual health in an Islamic country. The findings are organized into three main categories: Challenges, Opportunities, and Solutions. In the following sections, we will continue to discuss solutions to the challenges identified by exploring the associated opportunities.

### Structural challenges

The lack of human resources, structural features, software resources, and service referral pathways has resulted in difficult access to services, raising concerns about privacy and the confidentiality of information. Aligning with the country’s macro policies and adopting an appropriate management strategy at the university level can facilitate the development of physical infrastructure and the training of specialized personnel. In Howarth’s study, the components of comfortable services in the clinic were identified as privacy, the assurance of information confidentiality, and the provision of dignity-based services [17]. In another study, the low-barrier, “safe” nature of sexual health clinics facilitated clients’ ability to open up about concerns. The report highlights the need for privacy in sexual health clinics [18]. Several studies have reported that the design of a clinic can significantly contribute to creating an effective therapeutic atmosphere [19, 20]. Additionally, Ramchandani’s study emphasized the need to mobilize resources to support a stronger clinical infrastructure in specialized SHCs [21].

### Socio-cultural challenges

Research indicates that fear of stigma surrounding sexual issues has led to unmet needs for sexual health services, ultimately decreasing sexual health literacy among clients. Numerous studies have identified stigma as a significant barrier to visiting clinics or receiving specialized services [18, 21]. Ineffective communication increases the likelihood of misdiagnosis, which can lead to dissatisfaction between the therapist and the client. To address this challenge, we propose solutions that align with national policies and managerial interests, enhance coordination among departments, and support innovative services. Suggested measures include improving computer infrastructure to ensure confidentiality, offering research grants as incentives, and empowering service providers. In addition to emphasizing education and research, Medland considers the nature of these clinics to be innovative [12]. In this experience, two clinics with distinct approaches provided opportunities for patient autonomy. Policymakers and clients recommended leveraging national and social media to foster dialogue and promote the clinic. Additionally, using an application offers a more convenient and cost-effective way to access services. Support for campaigns such as “I’m getting tested for HIV” and youth and sexual health initiatives-like “student empowerment workshops on sexual self-care” by the Vice University for Cultural and Student Affairs-were initial steps towards de-stigmatization. In this context, Abdulai et al. stated that modern stigma management strategies involve making stigma a societal attribute by challenging, exposing, and normalizing it through web platforms [22, 23].

### Professional and organizational challenges

The high workload and dissatisfaction among specialists in this field have become apparent. Considering the identified opportunities, solutions such as empowering healthcare providers and optimizing the use of specialists have been proposed. In this context, Howarth et al. demonstrated that taking the time to engage with clients and address their concerns facilitated their comfort in visiting the clinic [17]. In Iran, many midwives have transitioned from their specialized roles to general healthcare provider positions. This shift toward unspecialized tasks has decreased their motivation to focus on clients’ sexual health. Furthermore, their high workload has restricted their ability to address sensitive topics, such as family sexual health issues. As a solution, a large group of midwives suggested during focus group discussions that midwifery roles be reinstated. Subsequently, reproductive health specialists have voiced their dissatisfaction with service fees and are advocating for insurance coverage. Improving satisfaction for both clients and specialists could encourage greater participation among their colleagues. Studies show that offering low-cost, anonymous services in clinics increases the likelihood of referrals, particularly among sexual minorities [21, 24].

### Service challenges

This category highlights gaps in current service packages, the lack of culturally specific offerings, and insufficient insurance coverage. Studies have reported that the attitudes and behaviors of healthcare providers are major institutional barriers. Judgmental attitudes, lack of skills, and breaches of privacy and confidentiality have been identified as significant impediments. Additionally, service utilization is further hindered by the physical unavailability of services, unwelcoming environments within health facilities, and shortages of medicines and medical supplies [24, 25]. A patient’s perception on the quality of care are often dependent on the experiences they have with their healthcare practitioners. Studies indicate that effective communication directly correlates with favorable health outcomes. Since communication is the primary method all medical personnel utilize to assess and manage a patient, its collapse becomes detrimental to the entire healthcare system.

This is why strategically deaf patients, patients with low English language skills, and those who need assistance rely on well-trained professional medical interpreters. Such persons constitute an integral part of the healthcare system as they fill the gaps in culture and language that exist between the patients, family, caregivers, and healthcare professionals [26].

Given the clinic’s alignment with national policies and the expertise available at the university, proposed solutions include developing an evidence-based service package and integrating it into the country’s basic services. As recommended in the Ford study, we should shift from focusing on morbidity to adopting an integrated, health-promoting approach to sexual health [27]. Also, Ramchandani recommended integrating STI care with HIV prevention and treatment programs [21]. Public clinics lack sufficient days to meet community needs, while private consultations can be costly. Reproductive health specialists emphasis the need for sexual health counseling services to be included in the relative values book and urge the Supreme Insurance Council to approve insurance coverage for these services. The results of Ravindran’s study confirm those of the current study [28]. In agreement with the present study, another study found that distance to clinics and associated costs created barriers to service access [29].

The study is one of the first to examine the experiences of establishing sexual health clinics in Iran, focusing on stakeholders’ perspectives regarding the challenges faced by two university-affiliated public clinics. It aimed to gather insights from gender and religious minorities, as well as policymakers involved in the clinics’ establishment, to identify potential improvement strategies. However, the study has qualitative limitations, highlighting the need for cultural adaptation when applying its findings. Future research is encouraged to include a broader range of religious minorities and immigrant populations.

## Conclusion

This qualitative study examines the challenges and opportunities present in sexual health clinics in Iran. It emphasizes the need for improved access, education, and resource allocation. By addressing structural, socio-cultural, and professional barriers, stakeholders can enhance service delivery and increase sexual health literacy. Future efforts should prioritize culturally sensitive policies, enhanced interdisciplinary collaboration, and evidence-based interventions to improve sexual health services in Iran.

### Implications

The findings highlight the importance of interdisciplinary collaboration and culturally sensitive approaches to meeting the diverse needs of various populations.

## Data Availability

No datasets were generated or analysed during the current study.

## Abbreviations

SHCs: Sexual health clinics
FGD: Focus group discussion
HPV: Human Pupiloma Virous
HIV: Human immunodeficiency virus
STIs: Sexually transmitted infections
